# Relationship between advanced lung cancer inflammation index and long-term all-cause, cardiovascular, and cancer mortality among type 2 diabetes mellitus patients: NHANES, 1999–2018

**DOI:** 10.3389/fendo.2023.1298345

**Published:** 2023-11-28

**Authors:** Yaying Chen, Mengqian Guan, Ruiqi Wang, Xuewen Wang

**Affiliations:** ^1^Department of Physical Examination Center, Xiamen Humanity Hospital of Fujian Medical University, Xiamen, Fujian, China; ^2^Fuzhou International Travel Health Care Center, Fuzhou, China; ^3^Department of Gastroenterology, Xiamen Humanity Hospital of Fujian Medical University, Xiamen, Fujian, China; ^4^Department of Histology and Embryology, School of Basic Medical Sciences, Fujian Medical University, Fuzhou, Fujian, China

**Keywords:** type 2 diabetes mellitus, advanced lung cancer inflammation index, National Health and Nutrition Examination Survey, all-cause mortality, CVD mortality, cancer mortality

## Abstract

**Background:**

Type 2 diabetes mellitus (T2DM) was a major global health threat. As a chronic low-grade inflammatory disease, the prognosis of diabetes was associated with inflammation. The advanced lung cancer inflammation index (ALI) served as a comprehensive index to assess inflammation. This study aimed to estimate the association between ALI and all-cause, cardiovascular disease (CVD), and cancer mortality in T2DM patients.

**Methods:**

We extracted cohort data from the National Health and Nutrition Examination Survey (NHANES) spanning 1999-2018 for analysis. The weighted Kaplan-Meier analysis and multivariate-adjusted Cox analysis were utilized to evaluate the relationship between ALI and all-cause, CVD, and cancer mortality in T2DM patients. Restricted cubic spline (RCS) analysis was employed to assess their non-linear relationship. Stratified analysis and interaction analysis were conducted to enhance the robustness of the results.

**Results:**

The study incorporated a total of 3,888 patients. An increase in ALI was associated with a reduced risk of all-cause and CVD mortality in T2DM patients, but not related to cancer mortality. There were J-shaped and L-shaped non-linear relationships between ALI and all-cause, CVD mortality in T2DM patients, respectively. The inflection points were 90.20 and 93.06, respectively. For values below the inflection point, every 10U increase in ALI, both all-cause and CVD mortality risk decreased by 9%. Beyond the inflection point, all-cause mortality rose by 3%, while CVD mortality remained unaffected. Gender-stratified RCS analysis indicated a linear negative relationship between CVD mortality and ALI in female T2DM patients, whereas the trend in males aligned with the overall population.

**Conclusion:**

Our research initially identified a significant correlation between increased ALI levels with decreased all-cause and CVD mortality in T2DM patients. There were J-shaped and L-shaped non-linear relationships between ALI and all-cause, CVD mortality in T2DM patients, respectively. For female patients, there was a linear negative relation between CVD mortality and ALI, whereas the trend in males aligned with the overall population. These findings suggested that maintaining ALI (for example, control body weight and keep albumin in the normal range) within a certain range in the clinical settings was crucial for improving all-cause and CVD mortality in T2DM patients.

## Introduction

Nowadays, approximately 529 million adults are afflicted with diabetes mellitus (DM) on the global scale in 2021, with over 90% having type 2 diabetes mellitus (T2DM). The global age-standardized total DM prevalence was 6.1% (1). Compared to patients without T2DM, 2 to 4 folds the risk of cardiovascular disease (CVD) and death was observed in patients with T2DM (2). Despite significant progress in DM treatment, DM remained one of the leading causes of death and disability worldwide, especially affecting all-cause and CVD mortality (1, 2). Consequently, identifying prognostic factors that may prevent or delay DM mortality was of paramount importance.

Previous studies have indicated that T2DM was fundamentally a chronic low-grade inflammatory disease, as evidenced by remarkable elevation of inflammatory factors such as serum interleukin-6 (IL-6), tumor necrosis factor (TNF), and C-reactive protein (CRP) (3–6). Hence, inflammation might play a crucial role in the occurrence and development of DM, ultimately decreasing the survival of patients with DM. Currently, most research regarding inflammatory markers for assessing the prognosis of DM was focused on a single factor. However, chronic inflammation could lead to adipocyte accumulation and insulin resistance through inflammatory factors such as TNF- α and CRP, thereby causing changes in body weight and albumin levels (3, 4). In addition, albumin level, obesity is associated with the development of DM and complications (7, 8). Thus, relying on a single inflammatory index might not provide sufficient accuracy to estimate the prognosis of patients with DM.

The advanced lung cancer inflammation index (ALI), including body mass index (BMI), albumin, and neutrophil to lymphocyte ratio (NLR), was a systemic inflammation index that was primarily applied in lung cancer patients (9–12). After that, ALI was utilized for other cancers, comprising esophageal, colorectal, pancreatic, and gastric cancer (13–16). Due to the comprehensive function of ALI in assessing inflammation, several studies have also explored the relationship between ALI and the prognosis of inflammation-related diseases such as hypertension, heart failure, and coronary artery disease (17–19). DM was considered to be associated with inflammation, however, the relationship between ALI and DM was still unknown. consequently, we firstly employed ALI to assess the impact on all-cause, CVD, and cancer mortality in patients with T2DM.

The National Health and Nutrition Examination Survey (NHANES), conducted by the National Center for Health Statistics (NCHS), was an extensive, multi-stage sampling study that was nationally representative and offered nutritional and health data for the American population. A cycle occurred every 2 years, surveying around 6,000 individuals in each cycle (20).

This study aimed to investigate the relationship between ALI levels and the risk of all-cause, CVD, and cancer mortality in T2DM patients. Additionally, we further quantified the impact of changes in ALI levels on long-term survival, thereby providing preliminary insights for the treatment and management of T2DM patients.

## Methods

### Study population

**Figure 1:**
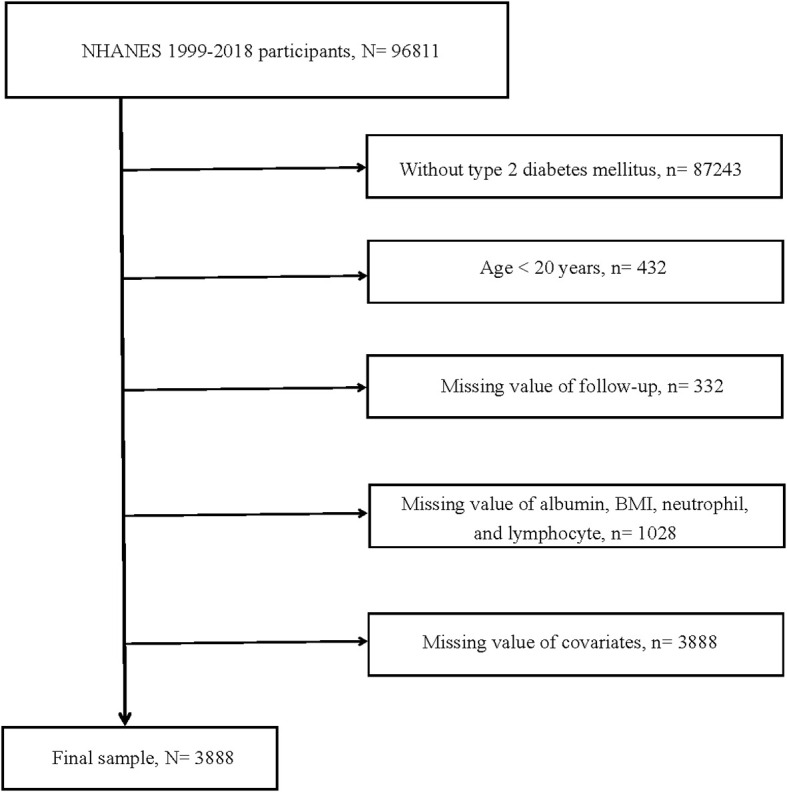
Flow chart of study participants. BMI, body mass index. N, The number of patients being included. n, The number of patients being excluded.

Public data from NHANES for the years 1999 to 2018 were collected for the current study. The data collection process and sampling methodology have been published elsewhere (21). Inclusion criteria for this research were as follows: 1) individuals diagnosed with T2DM; 2) age above 20 years. Exclusion criteria were as follows: 1) absence of data on survival status and follow-up time; 2) lack of data on albumin, lymphocytes, neutrophils, and BMI; 3) missing information on other covariates. The diagnosis of T2DM was based on the following index: 1) individuals have been told by doctor that they have DM; 2) fasting blood glucose levels ≥7.0mmol/L; 3) two-hour Oral Glucose Tolerance Test blood glucose levels ≥11.1mmol/L; 4) random blood glucose levels ≥11.1mmol/L; 5) glycated hemoglobin (HbA1c) ≥6.5%; 6) use of glucose-lowering medications or insulin; 7) exclusion of type 1 diabetes mellitus. Finally, a total of 3888 individuals over the age of 20 with T2DM were encompassed in the current study. **Figure 1** illustrated the detailed screening procedure for this study.

### Measurement of ALI

Due to the inflammation level, nutritional status, and body weight all have significant effects on diabetes, the ALI was used to comprehensively assess their effects on DM. ALI was composed of BMI, albumin, and NLR, with the specific formula being BMI (kg/m2) * albumin level (g/dL)/NLR. NLR was calculated as neutrophil counts divided by lymphocyte counts. According to the ALI levels, patients were divided into four groups based on quartile, namely the Quantile1 group, Quantile2 group, Quantile3 group, and Quantile4 group.

### Identification of mortality

The mortality of all-cause, CVD, and cancer was ascertained using data from the NCHS, referenced to the National Death Index as of 31 December 2019 (22). More specifically, CVD mortality was identified based on ICD-10 codes (I00–I078). Cancer mortality was confirmed as deaths from malignant neoplasms (C00-C97). The National Death Index was employed to determine individuals’ follow-up time by subtracting the baseline examination date from the latest known mortality status.

### Covariates definitions

Based on standardized questionnaires, we extracted data on the participants’ sociodemographic characteristics (age, sex, race, education level, and poverty income ratio (PIR)), smoking and drinking status, duration of DM, glucose-lowering medications or insulin usage for DM, and hypertension. Data on body weight and height were obtained from the mobile examination center. Additionally, Albumin, neutrophil counts, lymphocyte counts, alanine aminotransferase (ALT), hemoglobin A1c (HbA1c), and creatinine (Cr) were collected from laboratory measurements. The missing data for these covariates were excluded from the current study. The detailed definition and classification of the above covariates were shown in **Supplementary Methods**.

### Statistical analysis

Statistical analysis of the current study was rigorously conducted according to the design recommended by NHANES, taking into account sample weights, clustering, and stratified analysis. For continuous variables that conformed to a normal distribution, mean ± standard deviation was employed for representation, while those that did not were presented employing the median (25th percentile, 75th percentile). An analysis of variance was constructed to evaluate baseline differences in continuous variables. Baseline discrepancies in categorical variables were estimated using the χ2 test and were shown in the form of counts (percentages).

Kaplan-Meier analysis was employed for the preliminary investigation of the relationships among all-cause, CVD, and cancer mortality in ALI levels and patients with T2DM. After adjusting for multiple covariates, both univariate and multivariate Cox regression analyses were applied to further examine the influence of ALI levels on all-cause, CVD, and cancer mortality among patients with T2DM, with results presented as Hazard Ratio (HR) and 95% Confidence Interval (CI). A total of 4 models were utilized in this study as follows: The crude model, implying that no adjustments were made for confounders. Model 1 implied that adjustments were made for age, sex, race, education level, and PIR, which was used to control the impact of demographic factors. Model 2 indicated that adjustments were made for age, sex, race, education level, PIR, smoking status, alcohol status, hypertension, and medication usage, which was used to control the impact of demographic factors and a history of prior disease. Model 3 was adjusted for age, sex, race, education level, PIR, smoking status, alcohol status, hypertension, medication usage, ALT, HbA1c, and Cr. which was used to control the impact of demographic factors, a history of prior disease and laboratory indicators.

The aforementioned analyses were conducted employing data weighted with NHANES-recommended weights.

We integrated restricted cubic spline (RCS) analysis with the multivariate-adjusted COX regression model, for the purpose of evaluating the non-linear associations in all-cause, CVD, and cancer mortality between ALI levels and T2DM patients. When a non-linear relationship was present, we would employ a recursive algorithm to determine its inflection points. For further examination of threshold effects, we employed piecewise Cox regression models for evaluation. Moreover, after dividing the ALI levels of each participant by 10, we incorporated them as continuous variables into a multivariable Cox regression analysis, aiming to evaluate the impact of per 10-unit change in ALI on all-cause, CVD, and cancer mortality among T2DM patients. This approach aims to better quantify the impact of ALI level changes on the prognosis of T2DM patients, offering a more comprehensive insight (either in quantile ALI or per 10U increment of ALI). It was also advantageous for clinicians to dynamically assess the prognosis of T2DM patients based on ALI level.

To enhance the robustness of the current study, we conducted several sensitivity analyses. Initially, we established stratified analysis to estimate the potential interactions between ALI levels and stratification variables, which consisted of age (< 60 or ≥ 60 years), sex (male or female), race (White, Black, Mexican American or others), smoking status (never, former or now), hypertension (no or yes), use of glucose-lowering drugs or insulin, and HbA1c levels (< 7 or ≥ 7%). Moreover, taking into account that the duration of diabetes might affect the survival of T2DM patients, we re-conducted multi-model Cox analysis after excluding patients lacking information on the duration of diabetes.

All analyses were conducted using R software (version 4.3.1). Statistical significance was defined as a two-sided P < 0.05.

## Results

### Baseline characteristics

From the NHANES 1999-2018 cohorts, we initially extracted 96,811 participants, following the application of inclusion and exclusion criteria, a total of 3,888 participants were deemed eligible. The detailed flow was presented in **Figure 1**. Participants were categorized into four groups according to their ALI levels, each group consisting of 972 individuals. The average ALI values for Quantile1 group, Quantile2 group, Quantile3 group, and Quantile4 group were 33.02, 54.29, 73.68, and 121.83, respectively. The average age of these 3,888 participants was 60.03 years.

In comparison to the Quantile1 group, the Quantile2, Quantile3, and Quantile4 groups exhibited distinct characteristics: They are younger in terms of age; Their BMI values were higher; A higher percentage of females was observed in terms of gender composition; The proportion of White individuals was lower; There was a reduced percentage of individuals with diabetes duration exceeding 10 years; A higher percentage without using glucose-lowering drugs or insulin; Regarding laboratory indicators, there were higher levels of albumin, lymphocytes, and ALT, lower levels of neutrophils, NLR, and Cr. Similar education levels, PIR, HbA1c, smoking status, alcohol status, and hypertension status were observed. Detailed information was provided in **Table 1**.

**Table 1:** Baseline demographic and medical characteristics of patients with T2DM in the NHANES 1999–2018 cohort.

Characteristics	ALI					
	Total	Quantile 1 34.65 [2.83,44.94]	Quantile 2 54.29 (44.94,63.49]	Quantile 3 73.13 (63.49,87.28]	Quantile 4 111.78 (87.28,678.40]	*P*
Participants, n	3888	972	972	972	972	
ALI, mean	69.97 (67.95,72.00)	33.02 (32.33, 33.70)	54.29 (53.84, 54.74)	73.68 (73.04, 74.32)	121.83 (116.69,126.97)	< 0.0001
Age, year	60.03 (59.47,60.58)	64.74 (63.75,65.72)	61.03 (59.95,62.10)	58.22 (57.30,59.14)	55.97 (54.68,57.25)	< 0.0001
Gender, n (%)						< 0.0001
Female	1999(51.05)	611(60.61)	493(51.51)	485(51.41)	410(40.09)	
Male	1889(48.95)	361(39.39)	479(48.49)	487(48.59)	562(59.91)	
Race, n (%)						< 0.0001
White	1414(64.05)	455(72.71)	386(67.36)	318(61.29)	255(54.36)	
Black	931(13.52)	143(7.20)	193(11.32)	215(12.12)	380(24.11)	
Mexican American	810(9.14)	178(6.91)	218(8.82)	227(10.53)	187(10.31)	
Other	733(13.29)	196(13.19)	175(12.50)	212(16.06)	150(11.23)	
Education level						0.35
Less than high school	1341(23.05)	327(22.76)	348(21.83)	326(21.77)	340(26.10)	
High school or equivalent	860(25.15)	225(23.73)	202(24.10)	222(26.95)	211(25.80)	
College or above	1687(51.80)	420(53.51)	422(54.07)	424(51.28)	421(48.10)	
Family income- to-poverty ratio						0.59
<1.3	1372(24.79)	323(23.47)	378(25.50)	330(24.55)	341(25.67)	
[1.3,3.5)	1557(39.52)	400(38.60)	367(40.59)	391(37.53)	399(41.50)	
≥3.5	959(35.69)	249(37.93)	227(33.91)	251(37.92)	232(32.83)	
BMI, Kg/m2	33.06 (32.70,33.43)	29.64 (29.06,30.21)	32.21 (31.60,32.81)	34.33 (33.59,35.08)	36.21 (35.47,36.95)	< 0.0001
Albumin, g/dL	4.17 (4.15,4.18)	4.12 (4.09,4.15)	4.16 (4.13,4.19)	4.17 (4.15,4.20)	4.21 (4.18,4.24)	< 0.001
Neutrophil, K/uL	4.72 (4.65,4.80)	5.74 (5.58,5.90)	4.99 (4.86,5.13)	4.47 (4.37,4.58)	3.63 (3.52,3.74)	< 0.0001
Lymphocyte, K/uL	2.17 (2.13,2.22)	1.52 (1.48,1.57)	2.04 (1.99,2.08)	2.33 (2.28,2.39)	2.83 (2.70,2.96)	< 0.0001
NLR, mean	2.47 (2.41,2.54)	4.10 (3.95,4.25)	2.47 (2.42,2.52)	1.94 (1.91,1.98)	1.34 (1.30,1.37)	< 0.0001
ALT, U/L	27.18 (26.05,28.31)	26.34 (22.86,29.81)	26.64 (25.09,28.18)	26.88 (25.62,28.14)	28.97 (27.56,30.38)	0.04
HbA1c, %	7.24 (7.16,7.32)	7.17 (7.01,7.34)	7.15 (7.03,7.27)	7.42 (7.25,7.58)	7.20 (7.06,7.35)	0.06
Cr, umol/L	86.17 (84.19,88.15)	101.57 (94.95,108.18)	85.53 (82.67, 88.38)	80.79 (78.16, 83.41)	76.52 (74.79, 78.26)	< 0.0001
Smoke status, n (%)						0.05
Never	1926(47.98)	421(43.25)	463(46.86)	516(47.74)	526(54.46)	
Former	1345(35.93)	384(40.71)	346(36.99)	326(35.65)	289(30.05)	
Now	617(16.09)	167(16.04)	163(16.15)	130(16.61)	157(15.50)	
Alcohol, n (%)						0.04
Never	1781(39.80)	459(39.59)	452(40.20)	442(39.93)	428(39.45)	
Mild to moderate	1647(48.17)	426(51.84)	402(47.95)	419(48.20)	400(44.48)	
Heavy	85(7.4)	18(10.40)	24(9.78)	23(8.63)	20(9.43)	
Hypertension, n (%)						0.49
No	985(27.09)	232(27.18)	261(27.37)	265(29.02)	227(24.58)	
Yes	2903(72.91)	740(72.82)	711(72.63)	707(70.98)	745(75.42)	
Duration of diabetes*						0.003
<3 years	656(18.64)	138(19.31)	160(20.63)	163(20.64)	195(27.35)	
3-10 years	1339(34.24)	320(37.19)	313(37.93)	363(44.44)	343(40.82)	
>10 years	1387(32.49)	413(43.50)	373(41.43)	322(34.92)	279(31.83)	
Medication use						< 0.0001
No insulin or pills	844(23.86)	181(19.07)	208(24.24)	220(24.15)	235(28.16)	
Only diabetes pills	2145(54.26)	522(52.50)	542(54.03)	525(53.93)	556(56.73)	
Only insulin	405(9.67)	148(16.45)	99(9.25)	93(7.73)	65(5.09)	
Both insulin and diabetes pills	494(12.22)	121(11.97)	123(12.48)	134(14.19)	116(10.02)	

ALI, advanced lung cancer inflammation index; BMI, body mass index; NLR, neutrophil to Lymphocyte ratio; ALT, alanine aminotransferase; Cr, creatinine.

Values are weighted mean (IQR) for continuous variables or numbers (weighted %) for categorical variables. Wilcoxon rank-sum test was used for continuous variables, and chi-squared test with Rao & Scott’s second-order correction was used for categorical variables.

*A total of 506 patients without duration of diabetes data.

### Kaplan-Meier analysis

**Figure 2:**
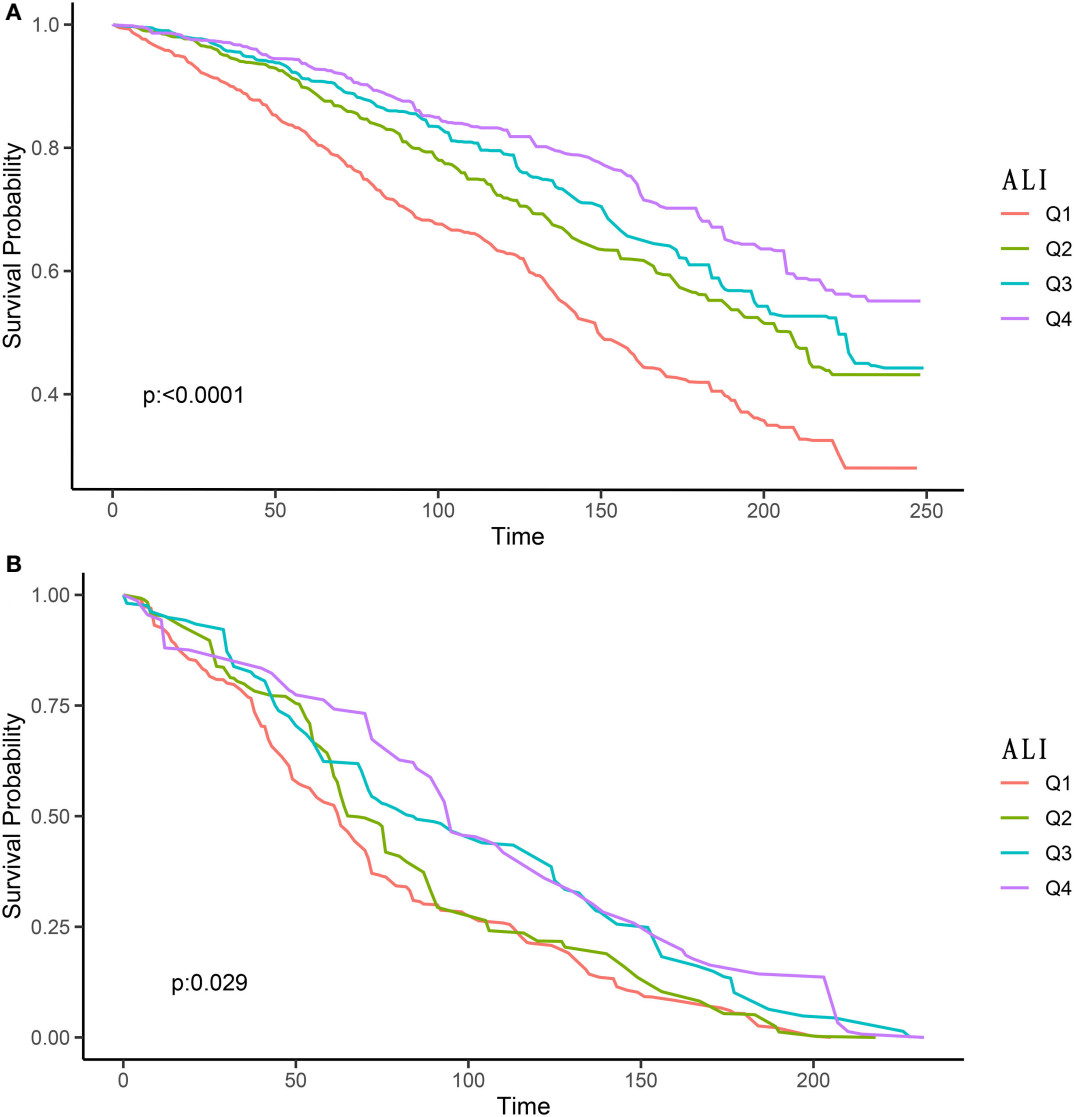
Kaplan-Meier survival curves of ALI impact on long-term all-cause **(A)** and CVD **(B)** mortality in patients with T2DM (weighted). ALI, advanced lung cancer inflammation index; CVD, cardiovascular disease; Q1, Quantile 1; Q2, Quantile 2; Q3, Quantile 3; Q4, Quantile 4.

Among 3,888 participants, a total of 1005 all-cause deaths, 320 CVD deaths, and 160 cancer deaths were documented. Kaplan-Meier analysis was utilized for the preliminary evaluation of the association between ALI levels and all-cause, CVD, and cancer mortality in T2DM patients. **Figure 2** illustrated that higher ALI levels were associated with decreased all-cause and CVD mortality in T2DM patients (P <0.0001, P =0.0029, respectively), while ALI levels were not correlated with cancer mortality (P =0.162), as shown in **Supplementary Figure S1**.

### ALI and mortality

Model 3 was a multivariable Cox analysis adjusted for age, gender, race, education level, PIR, smoking status, alcohol status, hypertension, use of glucose-lowering drugs or insulin, ALT, HbA1c, and Cr.

The findings suggested that higher ALI levels were associated with decreased all-cause mortality in T2DM patients. In comparison to the Quantile1 group, the HR (95% CI) for the Quantile2, Quantile3, and Quantile4 groups were 0.73 (0.58-0.92), 0.80 (0.61-1.04), and 0.76 (0.59-0.97), respectively (P_trend_ =0.04).

In CVD mortality, a similar trend was also observed, namely, higher levels of ALI correlate with lower CVD mortality in T2DM patients. The HR (95% CI) for the Quantile2, Quantile3, and Quantile4 groups were 0.86 (0.63-1.19), 0.73 (0.50-1.06), 0.68 (0.47-0.98), respectively (P_trend_ =0.04).

However, the trend of continuously decreasing HR was not found in cancer mortality, as indicated in **Table 2**. There was no correlation between ALI levels and cancer mortality, The HR (95% CI) for the Quantile2, Quantile3, and Quantile4 groups were 1.17 (0.64-2.17), 0.80 (0.46-1.40), 1.39 (0.61-3.18), respectively (P_trend_ =0.89).

**Table 2:** Relationships of ALI with all-cause, CVD, and cancer mortality in patients with T2DM from the NHANES 1999–2018 cohort.

ALI		All -cause mortality			
		Crude	Model 1	Model 2	Model 3
	No.death/total	HR, 95%CI	HR, 95%CI	HR, 95%CI	HR, 95%CI
Quantile 1	380/1005	ref	ref	ref	ref
Quantile 2	246/1005	0.62(0.48,0.80)	0.70(0.55,0.89)	0.70(0.55,0.88)	0.73(0.58,0.92)
Quantile 3	206/1005	0.51(0.40,0.65)	0.75(0.59,0.95)	0.76(0.58,0.99)	0.80(0.61,1.04)
Quantile 4	173/1005	0.41(0.31,0.53)	0.64(0.50,0.82)	0.70(0.55,0.90)	0.76(0.59,0.97)
Per 10 U increment		0.90(0.86,0.94)	0.96(0.92,0.99)	0.97(0.93,1.00)	0.97(0.94,1.01)
***P* for trend**		<0.0001	0.001	0.01	0.04
		CVD mortality			
		Crude	Model 1	Model 2	Model 3
		HR, 95%CI	HR, 95%CI	HR, 95%CI	HR, 95%CI
Quantile 1	128/320	ref	ref	ref	ref
Quantile 2	78/320	0.87(0.62,1.22)	0.80(0.57,1.10)	0.87(0.64,1.18)	0.86(0.63,1.19)
Quantile 3	64/320	0.61(0.42,0.89)	0.72(0.46,1.13)	0.71(0.49,1.04)	0.73(0.50,1.06)
Quantile 4	50/320	0.53(0.36,0.80)	0.61(0.44,0.85)	0.65(0.44,0.94)	0.68(0.47,0.98)
Per 10 U increment		0.92(0.87,0.98)	0.94(0.89,0.99)	0.94(0.89,1.00)	0.95(0.90,1.00)
***P* for trend**		0.002	0.01	0.03	0.04
		Cancer mortality			
		Crude	Model 1	Model 2	Model 3
		HR, 95%CI	HR, 95%CI	HR, 95%CI	HR, 95%CI
Quantile 1	57/160	ref	ref	ref	ref
Quantile 2	35/160	0.74(0.41,1.32)	0.92(0.51,1.65)	1.16(0.64,2.11)	1.17(0.64,2.17)
Quantile 3	35/160	0.54(0.33,0.89)	0.67(0.41,1.10)	0.72(0.45,1.16)	0.80(0.46,1.40)
Quantile 4	33/160	0.79(0.43,1.45)	1.14(0.55,2.35)	1.27(0.56,2.88)	1.39(0.61,3.18)
Per 10 U increment		0.96(0.90,1.02)	0.97(0.91,1.04)	0.97(0.91,1.04)	0.99(0.92,1.07)
***P* for trend**		0.16	0.73	0.81	0.89

ALI, advanced lung cancer inflammation index; ref, reference; HR, hazard ratios; CI, confidence interval; CVD, cardiovascular disease; ALT, alanine aminotransferase; Cr, creatinine;

Values are n or weighted HR (95% CI). Model 1: adjusted for age (years), gender (male or female), race or ethnicity (White, Black, Mexican American, or other), education levels (less than high school, high school or equivalent, or college or above), and family income-poverty ratio (<1.3, 1.3-3.5, ≥3.5). Model 2: model 1+ adjusted for smoke status (never, former, or now), alcohol (never, mild to moderate, or heavy), hypertension (yes or no), and medication use (no insulin or pills, only diabetes pills, only insulin or both insulin and diabetes pills). Model 3: model 2+ adjusted for ALT (U/L), HbA1c (%), and Cr (umol/L).

Evaluating the dynamic changes in ALI was critically important for the prognosis of T2DM patients. For each 10U increment in ALI, the multivariable-adjusted HR for all-cause mortality was 0.97 (0.94-1.01), for CVD mortality was 0.95 (0.90-1.00), for cancer mortality was 0.99 (0.92-1.07). The detailed results were exhibited in **Table 2**.

### Non-linear relationships

**Figure 3:**
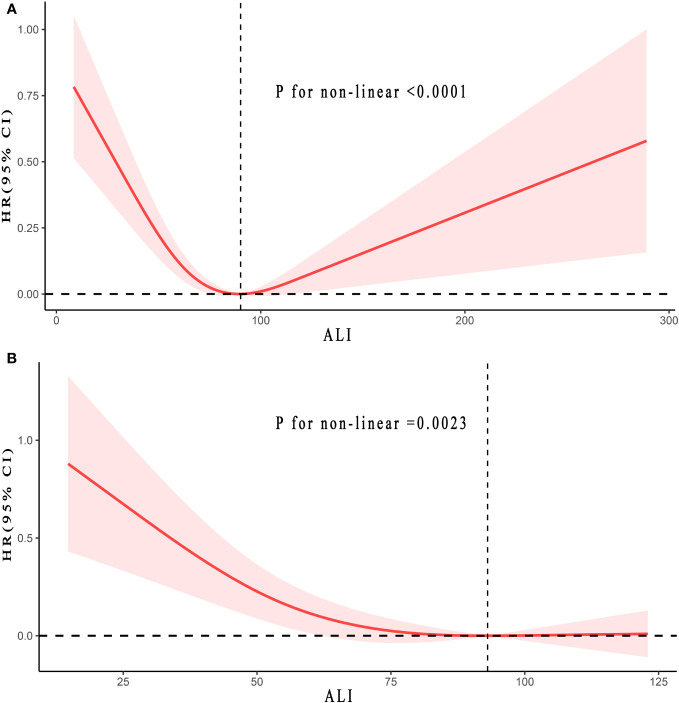
Relationship between ALI and all-cause **(A)** and CVD **(B)** mortality in patients with T2DM. Adjusted for age, gender, race, education levels, family income-poverty ratio, smoke status, alcohol, a history of hypertension, ALT, HbA1c (%), and Cr(umol/L). The solid and red shadow represent the estimated values and their 95% CIs, respectively. ALI, advanced lung cancer inflammation index; CVD, cardiovascular disease; ALT, alanine aminotransferase; Cr, creatinine. L-shaped non-linear relationships were observed in all Figures.

Based on Model 3, the RCS analysis indicated that a J-shaped non-linear relationship existed between ALI levels and all-cause mortality in T2DM patients (p < 0.0001), and an L-shaped non-linear relationship existed between ALI levels and CVD mortality (p = 0.0023). Nevertheless, the non-linear relationship was not found in terms of cancer mortality. The detailed findings were displayed in **Figure 3** and in **Supplementary Figure S2**.

This study revealed that the inflection points for all-cause mortality and CVD mortality were 90.20 and 93.06, respectively. For ALI values below 90.20, an increase of 10 U ALI corresponded to a 9% reduction in the all-cause mortality risk (HR: 0.91, 95% CI:0.86-0.97, P_trend_ =0.002). Yet, when ALI exceeded 90.20, every 10 U ALI increase led to a 3% rise in all-cause mortality risk (HR: 1.03, 95% CI:1.00-1.07, P_trend_ =0.04). For ALI values below 93.06, an increase of 10 U ALI corresponded to a 9% reduction in the CVD mortality risk (HR: 0.91, 95% CI:0.84-0.98, P_trend_ =0.02). However, when ALI exceeded 93.06, every 10 U ALI increase could not increase CVD mortality risk (HR: 1.01, 95% CI:0.98-1.04, P_trend_ =0.68). There was no inflection point for cancer mortality, hence, further analysis was not conducted. The details were presented in **Table 3**.

**Table 3:** Threshold effect analysis of ALI on all-cause, CVD mortality in patients with T2DM from the NHANES 1999–2018 cohort.

	All -cause mortality	
	Per 10U increment	*P*
<90.20	0.91(0.86,0.97)	0.002
>90.20	1.03(1.00,1.07)	0.04
	CVD mortality	
	Per 10U increment	*P*
<93.06	0.91(0.84,0.98)	0.02
>93.06	1.01(0.98, 1.04)	0.68

ALI, advanced lung cancer inflammation index; CVD, cardiovascular disease; ALT, alanine aminotransferase; Cr, creatinine.

Values are n or weighted HR (95% CI). Model is adjusted for age (years), gender (male or female), race or ethnicity (White, Black, Mexican American, or other), education levels (less than high school, high school or equivalent, or college or above), family income-poverty ratio (<1.3, 1.3-3.5, ≥3.5), smoke status (never, former, or now), alcohol (never, mild to moderate, or heavy), hypertension (yes or no), medication use (no insulin or pills, only diabetes pills, only insulin or both insulin and diabetes pills), ALT (U/L), HbA1c (%), and Cr(umol/L).

### Stratified and sensitivity analyses

The stratified analysis for potential interactions in all-cause mortality revealed no significant stratification variables. Similar results were also observed in CVD mortality.

Nevertheless, in cancer mortality, aside from race and smoking status, no significant interaction was seen in other stratification variables (**Supplementary Tables S1**–**S3**). Additionally, we evaluated whether there were interactions among the stratified variables after grouping by the inflection points of all-cause and CVD mortality. For all-cause mortality, the results indicated that except for gender, no significant interactions were observed for the other variables. In terms of CVD mortality, interactions were seen only for gender and smoking status. No significant interactions were observed for the rest (**Supplementary Tables S4**, **S5**).

For the female subgroup of patients, compared to the reference group (ALI <90.20 group and ALI <93.06 group, respectively), the all-cause mortality HR (95% CI) for the group with ALI exceeding 90.20 was 0.58 (0.43-0.78) (P_trend_ <0.001), and the HR (95%CI) for CVD mortality was 0.34 (0.19-0.61) (P_trend_ <0.001) for those with ALI greater than 93.06.

Consequently, we carried out an extra analysis on the non-linear relationships, segmented by gender. In terms of all-cause mortality, the results indicated that the trend for males and females was consistent with the overall population, which implied that as ALI increased, the mortality risk first decreased and then rose, with the inflection point being 83.65 and 103.15, respectively. The values of P for non-linear males and females were < 0.0001 and 0.04, respectively (**Supplementary Figure S3**).

Interestingly, there was a distinct difference in CVD mortality between males and females. Among males, prior to the inflection point (77.17), the risk of CVD mortality decreased with an increase in ALI (HR: 0.83, 95% CI:0.71-0.97, P =0.02). However, after the inflection point, there was no association between CVD mortality and ALI (HR: 1.11, 95% CI:0.99-1.24, P =0.09). Notably, in females, no inflection point was observed. A linear negative relationship existed between ALI and CVD mortality, as ALI increased, the risk of CVD mortality progressively dropped. With every 10U increase in ALI, the CVD mortality risk for female T2DM patients decreased by 11% (HR: 0.89, 95% CI:0.83-0.97, P =0.01). The results were displayed in **Supplementary Table S7** and **Supplementary Figure 4**.

Importantly, the sensitivity analysis revealed no remarkable changes in the results when excluding patients without information on the duration of diabetes. We incorporated an additional covariate (duration of diabetes) into the model 3 and re-performed the multivariable Cox analysis, as displayed in **Supplementary Table S6**.

## Discussion

This represented the inaugural research based on a broad cohort investigating the relationships between all-cause, CVD, and cancer mortality in ALI and T2DM patients. After multivariate adjustments, the increase in ALI was remarkably associated with reduced all-cause and CVD mortality, yet unrelated to cancer mortality. This study demonstrated that ALI has a J-shaped non-linear relationship with all-cause mortality and an L-shaped non-linear relationship with CVD mortality, with inflection points at 90.20 and 93.06, respectively. At values of ALI less than 90.20, every 10U increase in ALI decreased all-cause mortality risk by 9%. However, when it exceeded 90.20, every 10U increase in ALI instead enhanced the risk by 3%. For ALI values under 93.06, every 10U rise in ALI reduced the CVD mortality risk by 9%. Yet, when it’s above 90.20, an increase of 10U in ALI could not escalate the CVD mortality risk. Besides, non-linear relationship analysis stratified by gender revealed that both males and females displayed trends in line with the overall population concerning all-cause mortality, that was with the elevation of ALI, the mortality risk first declined then ascended, with distinct turning points at 83.65 for males and 103.15 for females. Whereas, regarding CVD mortality, males and females showcased vastly different patterns. Precisely, with rising ALI, females experienced a steady decline in CVD mortality risk, while males decreased to a certain point and then plateaus, with an inflection point at 77.17.

T2DM was a major global health threat, being the most common type of diabetes in adults and a lifelong metabolic disease. In 2021, about 67 million deaths were attributed to diabetes (23). Historically, diabetes has been viewed as an inflammation-related disease (3, 5, 24). Prolonged chronic inflammation disrupted the homeostasis of the pancreas and adipose tissue, affecting lipid metabolism and glucose absorption, leading to adipose tissue inflammation and insulin resistance, characterizing the nature of T2DM (3, 5). Chronic low-grade inflammation in diabetic patients could lead to atherosclerosis, thereby increasing the risk of CVD mortality (25, 26). Moreover, previous studies indicated that elevated level of inflammation was associated with an increased risk of diabetes-related mortality (27–29). All these results indicated that inflammation adversely affected the prognosis for T2DM patients, which was consistent with the results of our study. Nonetheless, it should be emphasized that previous research often relied on individual inflammatory markers to estimate the prognosis of T2DM patients, which was evidently insufficient for a comprehensive and accurate evaluation of the relationship between inflammation and the mortality risk of T2DM patients. One major factor was that individuals with T2DM frequently experienced malnutrition, which also played a significant role in the unfavorable outcomes of T2DM patients (30). Furthermore, inflammation could result in malnutrition, manifesting as decreased albumin levels and reduced BMI (31–33). Based on this, we contended that assessing the prognosis of T2DM patients should take into account both inflammation and malnutrition concurrently.

ALI was an index combining inflammation and nutrition, calculated by multiplying BMI by albumin and dividing by NLR. ALI was first applied to evaluate the mortality risk of patients with lung cancer (9–12). Subsequently, it was used for other cancers like esophageal, colorectal, pancreatic, stomach (13–16), and some inflammatory conditions like hypertension, heart failure, and coronary artery disease (17–19). No research has yet assessed the association between ALI and mortality in T2DM patients. Our research first demonstrated that among T2DM patients, a rise in ALI was remarkably linked to a decreased risk of all-cause and CVD mortality, but not significantly related to cancer mortality.

This study demonstrated that ALI presented a J-shaped and L-shaped non-linear relationship with all-cause and CVD mortality, respectively. The potential reasons could be analyzed from the following three dimensions. Firstly, NLR represented an immune-inflammatory response, and a high neutrophil count was a marker of non-specific inflammation. Conversely, a low lymphocyte count suggested a relative insufficiency in immune regulation (34). Therefore, an elevated NLR level could suggest the functional state of the immune system during chronic inflammation (35). Diabetes was a chronic low-grade inflammatory metabolic disease. Previous studies reported that patients with diabetes have a higher NLR level (35, 36), and the normal population’s average NLR ranged from 1.65 to 2.11 (37, 38). In this study, the average NLR for T2DM patients was 2.47. Activated leukocytes released reactive oxygen species via neutrophils and cytokines, thus promoting systemic inflammation and endothelial damage (39). Elevated NLR was associated with diabetic microvascular, macrovascular complications, and metabolic damage (35). All the above evidence suggested that the higher the NLR, the poorer the prognosis for T2DM individuals. Our study indicated that as groups moved from Quantile1 to Quantile4, NLR steadily dropped, and there was a corresponding decline in both all-cause and CVD mortality. Consequently, in the composite index ALI, we supposed that a lower NLR level chiefly contributed to a consistent reduction in all-cause and CVD mortality risks for T2DM patients.

Secondly, albumin was a commonly used nutritional assessment indicator. Prior studies indicated a negative correlation between albumin levels and the incidence of diabetes. Elevated levels of albumin could reduce the complications of diabetes, such as diabetic nephropathy, diabetic retinopathy, diabetic peripheral neuropathy, and the mortality associated with diabetes (7, 40, 41). Notably, albumin exhibited anti-inflammatory properties. Compared to the low albumin group, the levels of proinflammatory cytokines, such as TNF, and CRP, were significantly reduced in the high albumin group (42). In essence, elevated albumin levels could not only diminish the occurrence of diabetic complications, thereby enhancing the prognosis, but the anti-inflammatory attributed of albumin also contributed to a favorable prognosis. In this study, we noticed that from group Quantile1 to Quantile4, albumin levels gradually increased, and all-cause and CVD mortality progressively decreased. Consequently, we supposed that in the composite index ALI, elevated albumin levels mainly served to consistently decrease the all-cause and CVD mortality risks for T2DM patients.

Last but not least, the impact of BMI on the mortality of T2DM patients. A substantial amount of evidence indicated a positive correlation between BMI and the incidence of diabetes (43–45). Obesity promoted insulin resistance development, which could substantially increase the risk of type 2 diabetes mellitus. Besides, obesity was associated with increase in diabetes-related mortality (46). However, there was controversy over the effect of BMI on all-cause and CVD mortality in T2DM patients. Some studies reported that T2DM patients in higher BMI groups have a lower risk of mortality compared to those in the normal BMI group (47). Yet, other studies present a contrasting perspective. A meta-analysis incorporating 21 studies indicated that the lowest mortality risk was at a BMI value of 28.4 kg/m2. Using 28.4 as the threshold, for values below, the mortality risk decreased with an increase in BMI, while for values above, the mortality risk gradually increased with rising BMI (45). Another study involving 8,900 individuals indicated an inverse J-shaped non-linear relationship between BMI and mortality risk (44). This contradictory phenomenon might be explained by the obesity paradox. The obesity paradox referred to obesity being a risk factor for CVD events, yet patients with a high level of BMI tended to have better prognosis (48, 49). The underlying mechanism might be that patients with a high BMI possessed stronger anti-inflammatory effects and improved insulin resistance through the synthesis of adiponectin (50). Besides, the location of fat distribution was of paramount importance, compared to fat distributed in the viscera, subcutaneous fat was more favorable for prognosis (50). Last, patients with a high BMI possessed the ability of myocardial cells to produce vast protective antioxidative molecules when receiving stress signals from fat, which helped in preventing heart damage induced by obesity (51).

Based on the above, we contended that BMI has a more critical role in this non-linear association (there existed a J-shaped and L-shaped non-linear relationship between all-cause and CVD mortality in ALI and T2DM patients). Notably, the inflection points for all-cause and CVD mortality were 90.20 and 93.06, respectively. The study indicated that when ALI exceeded the inflection point, elevating ALI might not increase the risk of CVD mortality but could raise the risk of all-cause mortality. This suggested that in a clinical setting, we should be cautious with T2DM patients whose ALI values exceeded the inflection point. It’s essential to individually assess the impact of ALI on the prognosis of T2DM patients, facilitating the adoption of appropriate therapeutic interventions.

Previous study indicated that the mortality rate of T2DM patients in females was remarkably higher than in males, with a standard mortality rate of 1.46 for males and 1.72 for females, with females being 18% higher than males (52). Moreover, a meta-analysis showed that compared to males, females possessed a 13% increased risk of all-cause mortality and a 30% increased risk of CVD mortality among T2DM patients (53).

Firstly, the potential physiological mechanism might be attributed to the effects of estrogen. Before menopause, estrogen could play a protective role in female T2DM patients by increasing insulin sensitivity, while after menopause, the depletion of estrogen could increase the risk of CVD (54). Secondly, CVD was the primary cause of death in T2DM patients, accounting for over 50% of all deaths. Relative to males, females faced a greater risk of CVD mortality. One possible explanation was that, at the point of diagnosis, female patients tended to have more advanced arteriosclerosis (54). Interestingly, in the stratified analysis adjusted for multivariate, we observed that as ALI increased, the decrease in female mortality was significantly greater than in males, suggesting females were more likely to benefit from elevated ALI. This phenomenon might be attributed to the relationship between inflammation and diabetes-related deaths. Previous studies demonstrated that even mild glucose anomalies in female T2DM patients could lead to subclinical inflammation (54), resulting in arteriosclerosis and CVD mortality (25). Furthermore, this phenomenon was more pronounced after menopause, and the depletion of estrogen further intensified inflammation in T2DM female patients, worsening the prognosis (54). Conversely, testosterone could decrease inflammation levels in male T2DM patients, leading to an improved prognosis (53). Additionally, CVD was also an inflammatory disease, and elevated inflammation levels could increase the risk of CVD mortality (17). ALI could reflect the overall inflammatory level of the body, and an increase in ALI indicated a reduction in inflammation. Compared to males, females might have higher inflammation levels, and as ALI rose (indicating reduced inflammation), female patients could experience a more significant reduction in CVD mortality risk. In summary, we postulated that the key to extending the survival time of female T2DM patients lied in controlling inflammation levels. For male T2DM patients, inflammation needed to be controlled within an appropriate range. blindly reducing it might not yield additional survival benefits, indicating that other factors might influence the prognosis for male patients, such as the gut microbiota. This hypothesis required further validation through large-scale clinical trials.

Finally, although the findings suggested that cancer mortality was unrelated in ALI and T2DM patients, it was possible that this was due to too few patients with cancer-related mortality. Notably, previous studies showed that obesity was associated with an increased risk of cancer incidence and mortality. Intentional effective weight loss through surgical or nonsurgical means (novel diabetic medications such as glucagon-like peptide 1 agonists) might reduce cancer incidence and mortality (46, 55).

This study has several strengths. Firstly, our study, based on a large nationally representative sample, established a credible relationship between ALI and mortality in T2DM patients. Secondly, we considered a multitude of confounding factors, utilized multivariable-adjusted Cox analysis, stratified analysis, and interaction analysis to minimize biases and improve the reliability of the results. Furthermore, the sensitivity analysis, taking into account the duration of diabetes, yielded consistent findings. Lastly, ALI, serving as an easily calculable index for a comprehensive evaluation of inflammation, was highly convenient in clinical settings.

There were some limitations in this study, Firstly, this was an observational study. Despite the large sample size, it could not definitively establish a causal relationship between ALI and mortality in T2DM patients. The causality between ALI and mortality should be confirmed through interventional studies with large samples in the future. Secondly, even though we have employed as many methods as possible to eliminate biases, there still exist unknown confounding factors.

## Conclusions

Overall, our research initially identified a significant correlation between increased ALI levels with decreased all-cause and CVD mortality in T2DM patients. This study demonstrated that ALI has a J-shaped non-linear relationship with all-cause mortality and an L-shaped non-linear relationship with CVD mortality, with inflection points at 90.20 and 93.06, respectively. Notably, there were differences in CVD mortality between males and females. For female patients, there was a linear negative relation between CVD mortality and ALI, while in males, a peak effect was observed: when ALI surpasses 77.17, the risk of mortality would not decline with rising ALI. These findings suggested that maintaining ALI (for example, control body weight and keep albumin in the normal range) within a certain range in the clinical settings was crucial for improving all-cause and CVD mortality in T2DM patients. Individualized ALI value criteria based on gender could also maximize survival benefits for both male and female T2DM patients.

## Data availability statement

The raw data supporting the conclusions of this article will be made available by the authors, without undue reservation.

## Ethics statement

The studies involving humans were approved by the National Center for Health Statistics, CDC. The studies were conducted in accordance with the local legislation and institutional requirements. The participants provided their written informed consent to participate in this study.

## Author contributions

YC: Formal analysis, Methodology, Software, Writing – original draft. MG: Formal analysis, Methodology, Software, Writing – original draft. RW: Writing – review & editing, Methodology, Visualization. XW: Conceptualization, Formal analysis, Supervision, Validation, Writing – review & editing.
