# Supplementary material for: Relationship between advanced lung cancer inflammation index and long-term all-cause, cardiovascular, and cancer mortality among type 2 diabetes mellitus patients: NHANES, 1999–2018
Source: Front Endocrinol (Lausanne). 2023 Nov 28;14:1298345. doi: 10.3389/fendo.2023.1298345 (PMC10726345; doi:10.3389/fendo.2023.1298345)
Supplement: Supplementary file 1 [file DataSheet_1.doc]

**Supplementary Online Content**

**Supplementary Methods**

**Supplementary Table S1.** Stratified analyses of the relationships of ALI with all-cause mortality in patients with T2DM from the NHANES 1999–2018 cohort.

**Supplementary Table S2.** Stratified analyses of the relationships of ALI with CVD mortality in patients with T2DM from the NHANES 1999–2018 cohort.

**Supplementary Table S3.** Stratified analyses of the relationships of ALI with cancer mortality in patients with T2DM from the NHANES 1999–2018 cohort.

**Supplementary Table S4.** Stratified analyses of threshold effect about the relationships of ALI with all-cause mortality in patients with T2DM from the NHANES 1999–2018 cohort.

**Supplementary Table S5.** Stratified analyses of threshold effect about the relationships of ALI with CVD mortality in patients with T2DM from the NHANES 1999–2018 cohort.

**Supplementary Table S6** Relationships of ALI with all-cause, CVD, and cancer mortality in patients with T2DM from the NHANES 1999–2018 cohort, excluding the patients without duration of diabetes (n=3382).

**Supplementary Table S7.** Threshold effect analysis stratified by gender of ALI on CVD mortality in patients with T2DM from the NHANES 1999–2018 cohort.

**Supplementary Figure S1.** Kaplan-Meier survival curves of ALI impact on long-term cancer mortality in patients with T2DM (weighted).

**Supplementary Figure S2.** Relationship between ALI and cancer mortality in patients with T2DM.

**Supplementary Figure S3.** Relationship between ALI and all-cause mortality stratified by gender in patients with T2DM.

**Supplementary Figure S4.** Relationship between ALI and CVD mortality stratified by gender in patients with T2DM.

**Supplementary Methods**

Races were categorized into White, Black, Mexican American, or others. BMI was calculated by dividing weight in kilograms by height in meters squared. Regarding smoking status, it was grouped as never-smoker, former-smoker, or now-smoker, per their responses to whether they currently smoked and had spent at least 100 cigarettes in their life. Alcohol consumption was grouped by alcohol intake. The participants who consumed less than twelve drinks in a lifetime was categorized as a never drinker; those who consumed one to two drinks for female or one to two for male was categorized as a mild-moderate drinker; those who consumed more than two drinks for female and three drinks for male was categorized as a heavy drinker. Having a history of hypertension is grouped as taking anti-hypertensive medication for hypertension or a mean systolic blood pressure (SBP) greater than or equal to 140 mmHg and/or a mean diastolic blood pressure (DBP) greater than or equal to 90 mmHg when SBP and DBP were collected at MEC or self-reporting of doctor's diagnosis of hypertension. The duration of diabetes was calculated by subtracting the age at diagnosis from the current age and was divided into three groups: <3 years, 3-10 years, and >10 years. Medication use was divided into four groups based on whether antidiabetic drugs or insulin were used: No insulin or pills, Only diabetes pills, Only insulin, Both insulin and diabetes pills.

**Supplementary Table S1. Stratified analyses of the relationships of ALI with all-cause mortality in patients with T2DM from the NHANES 1999–2018 cohort**

| **Characteristics** | **ALI** | | | | | |
| --- | --- | --- | --- | --- | --- | --- |
|  | **Quantile 1**  34.65  [2.83,44.94] | **Quantile 2**  54.29  (44.94,63.49] | **Quantile 3**  73.13  (63.49,87.28] | **Quantile 4**  111.78  (87.28,678.40] | ***P* for trend** | ***P* for interaction** |
| Participants, n | 972 | 972 | 972 | 972 |  |  |
| Age |  |  |  |  |  | 0.44 |
| <60 | ref | 0.50(0.26,0.98) | 0.71(0.42,1.19) | 0.66(0.36,1.20) | 0.39 |  |
| ≥60 | ref | 0.71(0.55,0.93) | 0.66(0.50,0.88) | 0.55(0.41,0.74) | <0.0001 |  |
| Gender |  |  |  |  |  | 0.26 |
| Male | ref | 0.63(0.46,0.86) | 0.69(0.49,0.96) | 0.73(0.51,1.06) | 0.05 |  |
| Female | ref | 0.72(0.50,1.04) | 0.68(0.44,1.03) | 0.48(0.34,0.69) | <0.001 |  |
| Race |  |  |  |  |  | 0.13 |
| White | ref | 0.66(0.48,0.91) | 0.72(0.52,0.99) | 0.62(0.44,0.86) | 0.01 |  |
| Black | ref | 0.59(0.42,0.82) | 0.57(0.38,0.85) | 0.36(0.24,0.52) | <0.0001 |  |
| Mexican  American | ref | 0.41(0.23,0.72) | 0.48(0.30,0.77) | 0.53(0.30,0.95) | 0.03 |  |
| Other | ref | 1.10(0.46,2.65) | 0.51(0.20,1.30) | 0.66(0.28,1.56) | 0.15 |  |
| Smoke status |  |  |  |  |  | 0.27 |
| Never | ref | 0.70(0.47,1.04) | 0.86(0.60,1.23) | 0.66(0.44,0.99) | 0.1 |  |
| Former | ref | 0.58(0.40,0.83) | 0.47(0.32,0.69) | 0.49(0.34,0.71) | <0.0001 |  |
| Now | ref | 0.82(0.52,1.30) | 0.90(0.49,1.63) | 0.62(0.35,1.10) | 0.19 |  |
| Hypertension |  |  |  |  |  | 0.13 |
| No | ref | 0.46(0.26,0.82) | 0.71(0.50,1.03) | 0.37(0.19,0.74) | 0.004 |  |
| Yes | ref | 0.72(0.55,0.95) | 0.68(0.51,0.89) | 0.62(0.47,0.82) | <0.001 |  |
| Medication use |  |  |  |  |  | 0.40 |
| No insulin or  pills | ref | 0.54(0.30,0.97) | 0.49(0.26,0.92) | 0.32(0.16,0.62) | 0.002 |  |
| Only diabetes  pills | ref | 0.72(0.53,0.97) | 0.76(0.55,1.06) | 0.64(0.45,0.91) | 0.02 |  |
| Only insulin | ref | 0.62(0.36,1.05) | 0.75(0.40,1.40) | 0.88(0.52,1.47) | 0.48 |  |
| Both insulin  and diabetes pills | ref | 0.91(0.44,1.89) | 0.66(0.34,1.27) | 0.47(0.22,1.00) | 0.03 |  |
| HbA1c |  |  |  |  |  | 0.53 |
| <7 | ref | 0.63(0.45,0.87) | 0.59(0.42,0.81) | 0.51(0.36,0.72) | <0.0001 |  |
| ≥7 | ref | 0.73(0.52,1.01) | 0.78(0.55,1.10) | 0.63(0.43,0.95) | 0.04 |  |

**Supplementary Table S2. Stratified analyses of the relationships of ALI with CVD mortality in patients with T2DM from the NHANES 1999–2018 cohort**

| **Characteristics** | **ALI** | | | | | |
| --- | --- | --- | --- | --- | --- | --- |
|  | **Quantile 1**  34.65  [2.83,44.94] | **Quantile 2**  54.29  (44.94,63.49] | **Quantile 3**  73.13  (63.49,87.28] | **Quantile 4**  111.78  (87.28,678.40] | ***P* for trend** | ***P* for interaction** |
| Participants, n | 972 | 972 | 972 | 972 |  |  |
| Age |  |  |  |  |  | 0.88 |
| <60 | ref | 2.61(0.72,9.47) | 0.99(0.38,2.53) | 0.33(0.04, 2.75) | 0.23 |  |
| ≥60 | ref | 0.81(0.59,1.11) | 0.64(0.40,1.03) | 0.50(0.34,0.74) | <0.0001 |  |
| Gender |  |  |  |  |  | 0.06 |
| Male | ref | 1.03(0.67,1.59) | 0.83(0.52,1.31) | 0.89(0.58,1.35) | 0.35 |  |
| Female | ref | 0.75(0.39,1.44) | 0.52(0.33,0.84) | 0.31(0.15,0.64) | <0.001 |  |
| Race |  |  |  |  |  | 0.25 |
| White | ref | 0.83(0.54,1.27) | 0.68(0.46,1.03) | 0.46(0.27,0.79) | 0.01 |  |
| Black | ref | 1.87(1.01,3.45) | 0.93(0.50,1.75) | 0.60(0.28,1.27) | 0.12 |  |
| Mexican  American | ref | 0.32(0.08,1.26) | 0.35(0.13,0.94) | 0.34(0.11, 1.06) | 0.03 |  |
| Other | ref | 0.44(0.04,4.96) | 0.07(0.00,2.19) | 0.10(0.00, 3.46) | 0.05 |  |
| Smoke status |  |  |  |  |  | 0.30 |
| Never | ref | 0.79(0.46,1.35) | 0.68(0.40,1.15) | 0.38(0.21,0.69) | 0.001 |  |
| Former | ref | 0.92(0.59,1.46) | 0.41(0.20,0.84) | 0.65(0.43,1.00) | 0.003 |  |
| Now | ref | 1.24(0.57,2.68) | 1.82(0.78,4.25) | 2.07(0.63, 6.86) | 0.13 |  |
| Hypertension |  |  |  |  |  | 0.48 |
| No | ref | 0.40(0.15,1.01) | 0.99(0.40,2.46) | 0.10(0.01, 1.10) | 0.07 |  |
| Yes | ref | 0.96(0.66,1.41) | 0.74(0.55,0.99) | 0.61(0.38,0.99) | 0.02 |  |
| Medication use |  |  |  |  |  | 0.10 |
| No insulin or  pills | ref | 0.37(0.13,1.07) | 0.54(0.18,1.58) | 0.32(0.05, 1.84) | 0.29 |  |
| Only diabetes  pills | ref | 0.89(0.54,1.46) | 0.89(0.54,1.46) | 0.57(0.37,0.88) | 0.04 |  |
| Only insulin | ref | 1.00(0.49,2.04) | 0.35(0.17,0.72) | 0.35(0.08,1.44) | 0.01 |  |
| Both insulin  and diabetes pills | ref | 1.06(0.09,11.80) | 0.61(0.03,11.46) | 1.23(0.06,24.39) | 0.94 |  |
| HbA1c |  |  |  |  |  | 0.88 |
| <7 | ref | 0.84(0.53,1.31) | 0.70(0.40,1.23) | 0.41(0.26,0.65) | <0.0001 |  |
| ≥7 | ref | 1.01(0.74,1.38) | 0.61(0.35,1.07) | 0.64(0.23,1.76) | 0.19 |  |

**Supplementary Table S3. Stratified analyses of the relationships of ALI with cancer mortality in patients with T2DM from the NHANES 1999–2018 cohort**

| **Characteristics** | **ALI** | | | | | |
| --- | --- | --- | --- | --- | --- | --- |
|  | **Quantile 1**  34.65  [2.83,44.94] | **Quantile 2**  54.29  (44.94,63.49] | **Quantile 3**  73.13  (63.49,87.28] | **Quantile 4**  111.78  (87.28,678.40] | ***P* for trend** | ***P* for interaction** |
| Participants, n | 972 | 972 | 972 | 972 |  |  |
| Age |  |  |  |  |  | 0.24 |
| <60 | ref | 0.13(0.01,1.72) | 0.00(0.00,0.62) | 0.03(0.00,0.50) | 0.30 |  |
| ≥60 | ref | 0.84(0.47,1.49) | 0.56(0.34,0.91) | 0.92(0.49,1.73) | 0.15 |  |
| Gender |  |  |  |  |  | 0.08 |
| Male | ref | 0.86(0.45,1.65) | 0.35(0.17,0.72) | 0.91(0.37,2.20) | 0.15 |  |
| Female | ref | 0.62(0.23,1.67) | 1.01(0.43,2.37) | 0.56(0.24,1.29) | 0.32 |  |
| Race |  |  |  |  |  | 0.02 |
| White | ref | 0.66(0.32,1.34) | 0.40(0.23,0.72) | 1.22(0.58,2.56) | 0.38 |  |
| Black | ref | 2.48(0.66, 9.30) | 0.69(0.22,2.13) | 0.44(0.16, 1.24) | 0.02 |  |
| Mexican  American | ref | 0.07(0.01, 0.51) | 0.46(0.04,4.63) | 0.24(0.03, 2.15) | 0.23 |  |
| Other | ref | 0.32(0.04, 2.73) | 0.02(0.00,2.94) | 0.01(0.00, 1.26) | 0.02 |  |
| Smoke status |  |  |  |  |  | 0.04 |
| Never | ref | 0.72(0.29,1.83) | 0.91(0.44,1.86) | 0.56(0.11,2.95) | 0.65 |  |
| Former | ref | 1.00(0.40,2.50) | 0.22(0.11,0.44) | 1.07(0.48,2.39) | 0.33 |  |
| Now | ref | 0.85(0.25, 2.85) | 0.40(0.02,7.20) | 0.44(0.12, 1.68) | 0.11 |  |
| Hypertension |  |  |  |  |  | 0.16 |
| No | ref | 0.27(0.01, 6.09) | 0.13(0.02,1.04) | 1.55(0.09,27.43) | 0.49 |  |
| Yes | ref | 0.83(0.47,1.48) | 0.62(0.34,1.12) | 0.81(0.41,1.60) | 0.23 |  |
| HbA1c |  |  |  |  |  | 0.30 |
| <7 | ref | 0.80(0.45,1.42) | 0.65(0.28,1.50) | 0.88(0.38,2.03) | 0.53 |  |
| ≥7 | ref | 1.50(0.61,3.68) | 0.40(0.17,0.90) | 0.82(0.33,2.05) | 0.13 |  |

**Supplementary Table S4. Stratified analyses of threshold effect about the relationships of ALI with all-cause mortality in patients with T2DM from the NHANES 1999–2018 cohort**

| **Characteristics** | **ALI** | | | |
| --- | --- | --- | --- | --- |
|  | <90.20 | >90.20 | ***P* for trend** | ***P* for interaction** |
| Age |  |  |  | 0.79 |
| <60 | ref | 0.87(0.54,1.41) | 0.58 |  |
| ≥60 | ref | 0.70(0.54,0.91) | 0.01 |  |
| Gender |  |  |  | 0.02 |
| Male | ref | 1.02(0.73,1.42) | 0.92 |  |
| Female | ref | 0.58(0.43,0.78) | <0.001 |  |
| Race |  |  |  | 0.57 |
| White | ref | 0.77(0.59,1.00) | 0.05 |  |
| Black | ref | 0.57(0.41,0.78) | <0.001 |  |
| Mexican  American | ref | 0.89(0.55,1.45) | 0.65 |  |
| Other | ref | 0.80(0.36,1.75) | 0.57 |  |
| Smoke status |  |  |  | 0.76 |
| Never | ref | 0.75(0.54,1.04) | 0.08 |  |
| Former | ref | 0.76(0.54,1.07) | 0.12 |  |
| Now | ref | 0.68(0.35,1.32) | 0.26 |  |
| Hypertension |  |  |  | 0.47 |
| No | ref | 0.65(0.35,1.18) | 0.16 |  |
| Yes | ref | 0.74(0.60,0.92) | 0.01 |  |
| Medication use |  |  |  | 0.30 |
| No insulin or  pills | ref | 0.49(0.27,0.89) | 0.02 |  |
| Only diabetes  pills | ref | 0.76(0.57,1.01) | 0.06 |  |
| Only insulin | ref | 0.95(0.60,1.53) | 0.84 |  |
| Both insulin  and diabetes pills | ref | 0.67(0.38,1.17) | 0.16 |  |
| HbA1c |  |  |  | 0.32 |
| <7 | ref | 0.67(0.48,0.91) | 0.01 |  |
| ≥7 | ref | 0.79(0.58,1.07) | 0.13 |  |

**Supplementary Table S5. Stratified analyses of threshold effect about the relationships of ALI with CVD mortality in patients with T2DM from the NHANES 1999–2018 cohort**

| **Characteristics** | **ALI** | | | |
| --- | --- | --- | --- | --- |
|  | <93.06 | >93.06 | ***P* for trend** | ***P* for interaction** |
| Age |  |  |  | 0.98 |
| <60 | ref | 0.27(0.06,1.20) | 0.09 |  |
| ≥60 | ref | 0.53(0.36,0.77) | 0.001 |  |
| Gender |  |  |  | <0.001 |
| Male | ref | 1.24(0.76,2.04) | 0.39 |  |
| Female | ref | 0.34(0.19,0.61) | <0.001 |  |
| Race |  |  |  | 0.67 |
| White | ref | 0.50(0.30,0.83) | 0.01 |  |
| Black | ref | 0.50(0.24,1.05) | 0.07 |  |
| Mexican  American | ref | 0.52(0.10,2.60) | 0.42 |  |
| Other | ref | 2.90(0.20,42.53) | 0.44 |  |
| Smoke status |  |  |  | 0.03 |
| Never | ref | 0.35(0.20,0.61) | <0.001 |  |
| Former | ref | 0.93(0.60,1.43) | 0.73 |  |
| Now | ref | 2.57(0.79, 8.37) | 0.12 |  |
| Hypertension |  |  |  | 0.45 |
| No | ref | 1.44(0.41, 5.09) | 0.57 |  |
| Yes | ref | 0.60(0.38,0.93) | 0.02 |  |
| Medication use |  |  |  | 0.07 |
| No insulin or  pills | ref | 0.60(0.18, 1.97) | 0.4 |  |
| Only diabetes  pills | ref | 0.70(0.45,1.08) | 0.11 |  |
| Only insulin | ref | 0.30(0.07,1.26) | 0.1 |  |
| Both insulin  and diabetes pills | ref | 1.34(0.30, 5.93) | 0.7 |  |
| HbA1c |  |  |  | 0.80 |
| <7 | ref | 0.45(0.27,0.75) | 0.002 |  |
| ≥7 | ref | 0.69(0.31,1.56) | 0.37 |  |

**Supplementary Table S6. Relationships of ALI with all-cause, CVD, and cancer mortality in patients with T2DM from the NHANES 1999–2018 cohort, excluding the patients without duration of diabetes (n=3382)**

| **ALI** | **All -cause mortality** | | | |
| --- | --- | --- | --- | --- |
|  | **Crude** | **Model 1** | **Model 2** | **Model 3** |
|  | **HR, 95%CI** | **HR, 95%CI** | **HR, 95%CI** | **HR, 95%CI** |
| Quantile 1 | ref | ref | ref | ref |
| Quantile 2 | 0.65(0.50,0.84) | 0.73(0.57,0.93) | 0.71(0.55,0.91) | 0.74(0.58,0.95) |
| Quantile 3 | 0.53(0.41,0.67) | 0.77(0.60,0.99) | 0.77(0.58,1.01) | 0.81(0.62,1.07) |
| Quantile 4 | 0.43(0.33,0.56) | 0.65(0.50,0.84) | 0.71(0.55,0.92) | 0.76(0.59,0.98) |
| ***P* for trend** | <0.0001 | 0.002 | 0.01 | 0.05 |
|  | **CVD mortality** | | | |
|  | **Crude** | **Model 1** | **Model 2** | **Model 3** |
|  | **HR, 95%CI** | **HR, 95%CI** | **HR, 95%CI** | **HR, 95%CI** |
| Quantile 1 | ref | ref | ref | ref |
| Quantile 2 | 0.93(0.65,1.32) | 0.85(0.61,1.19) | 0.92(0.68,1.26) | 0.93(0.66,1.31) |
| Quantile 3 | 0.65(0.44,0.95) | 0.76(0.49,1.19) | 0.72(0.46,1.12) | 0.75(0.49,1.14) |
| Quantile 4 | 0.53(0.35,0.79) | 0.60(0.43,0.85) | 0.65(0.44,0.96) | 0.69(0.47,1.00) |
| ***P* for trend** | 0.003 | 0.01 | 0.04 | 0.06 |
|  | **Cancer mortality** | | | |
|  | **Crude** | **Model 1** | **Model 2** | **Model 3** |
|  | **HR, 95%CI** | **HR, 95%CI** | **HR, 95%CI** | **HR, 95%CI** |
| Quantile 1 | ref | ref | ref | ref |
| Quantile 2 | 0.65(0.34,1.23) | 0.76(0.40,1.45) | 0.93(0.50,1.73) | 0.98(0.50,1.92) |
| Quantile 3 | 0.51(0.29,0.88) | 0.57(0.33,0.96) | 0.47(0.28,0.77) | 0.52(0.28,0.98) |
| Quantile 4 | 0.87(0.50,1.53) | 1.06(0.50,2.24) | 1.20(0.57,2.53) | 1.29(0.59,2.80) |
| ***P* for trend** | 0.3 | 0.63 | 0.49 | 0.77 |

Values are n or weighted HR (95% CI). Model is adjusted for age (years), gender (male or female), race or ethnicity (White, Black, Mexican American, or other), education levels (less than high school, high school or equivalent, or college or above), family income-poverty ratio (<1.3, 1.3-3.5, ≥3.5), smoke status (never, former, or now), alcohol (never, mild to moderate, or heavy), hypertension (yes or no), medication use (no insulin or pills, only diabetes pills, only insulin or both insulin and diabetes pills), duration of diabetes (<3 years, 3-10 years, and >10years), ALT (U/L), HbA1c (%), and Cr(umol/L).

**Supplementary Table 7. Threshold effect analysis stratified by gender of ALI on CVD mortality in patients with T2DM from the NHANES 1999–2018 cohort.**

|  | **CVD mortality of males** | |
| --- | --- | --- |
|  | **Per 10U increment** | ***P*** |
| <77.17 | 0.83(0.71,0.97) | 0.02 |
| >77.17 | 1.11(0.99,1.24) | 0.09 |
| Without  inflection  point | **CVD mortality of females** | |
|  | **Per 10U increment** | ***P*** |
|  | 0.89(0.83, 0.97) | 0.01 |

Values are n or weighted HR (95% CI). Model is adjusted for age (years), race or ethnicity (White, Black, Mexican American, or other), education levels (less than high school, high school or equivalent, or college or above), family income-poverty ratio (<1.3, 1.3-3.5, ≥3.5), smoke status (never, former, or now), alcohol (never, mild to moderate, or heavy), hypertension (yes or no), medication use (no insulin or pills, only diabetes pills, only insulin or both insulin and diabetes pills), ALT (U/L), HbA1c (%), and Cr(umol/L).


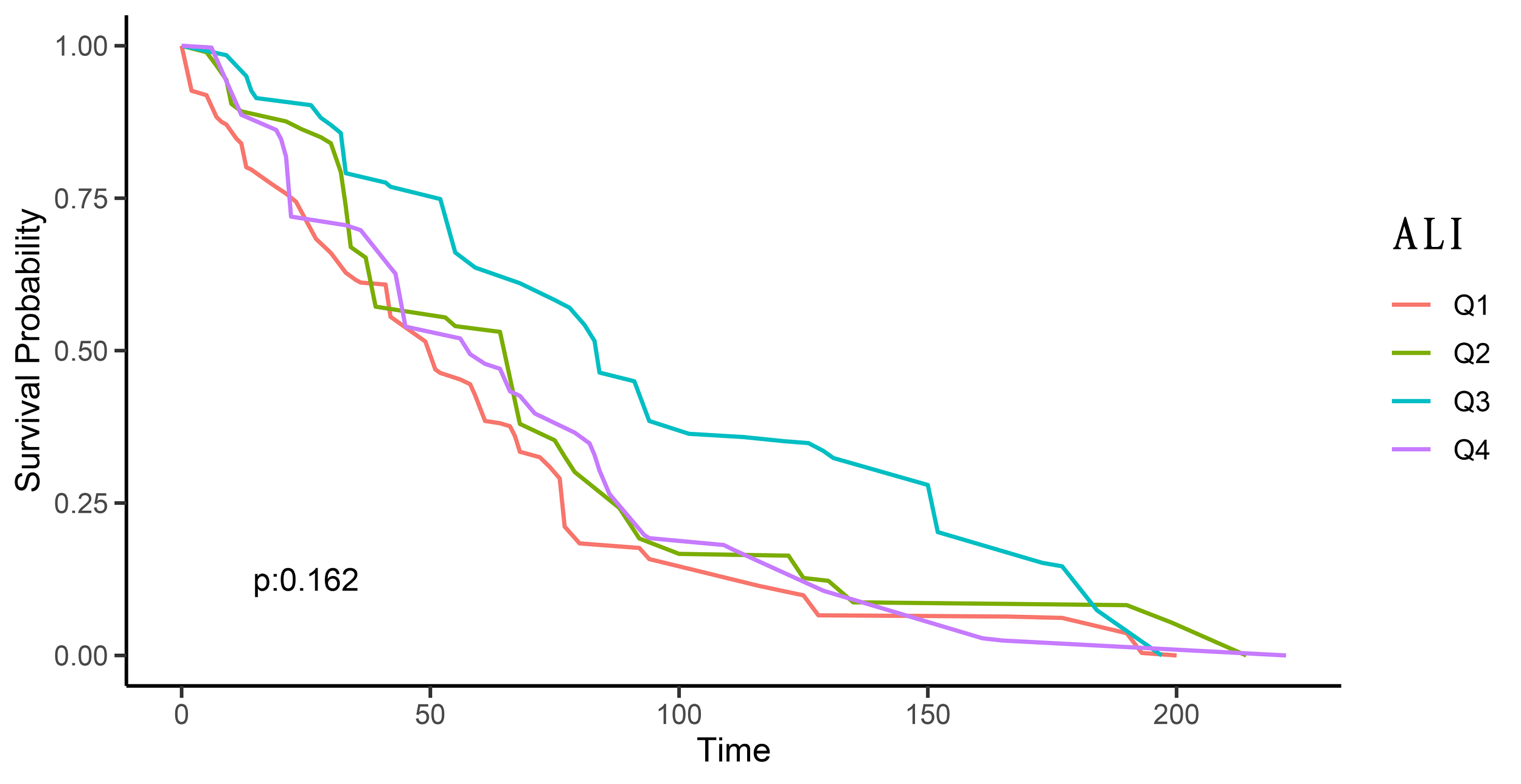


**Supplementary Figure S1.** Kaplan-Meier survival curves of ALI impact on long-term cancer mortality in patients with T2DM (weighted).


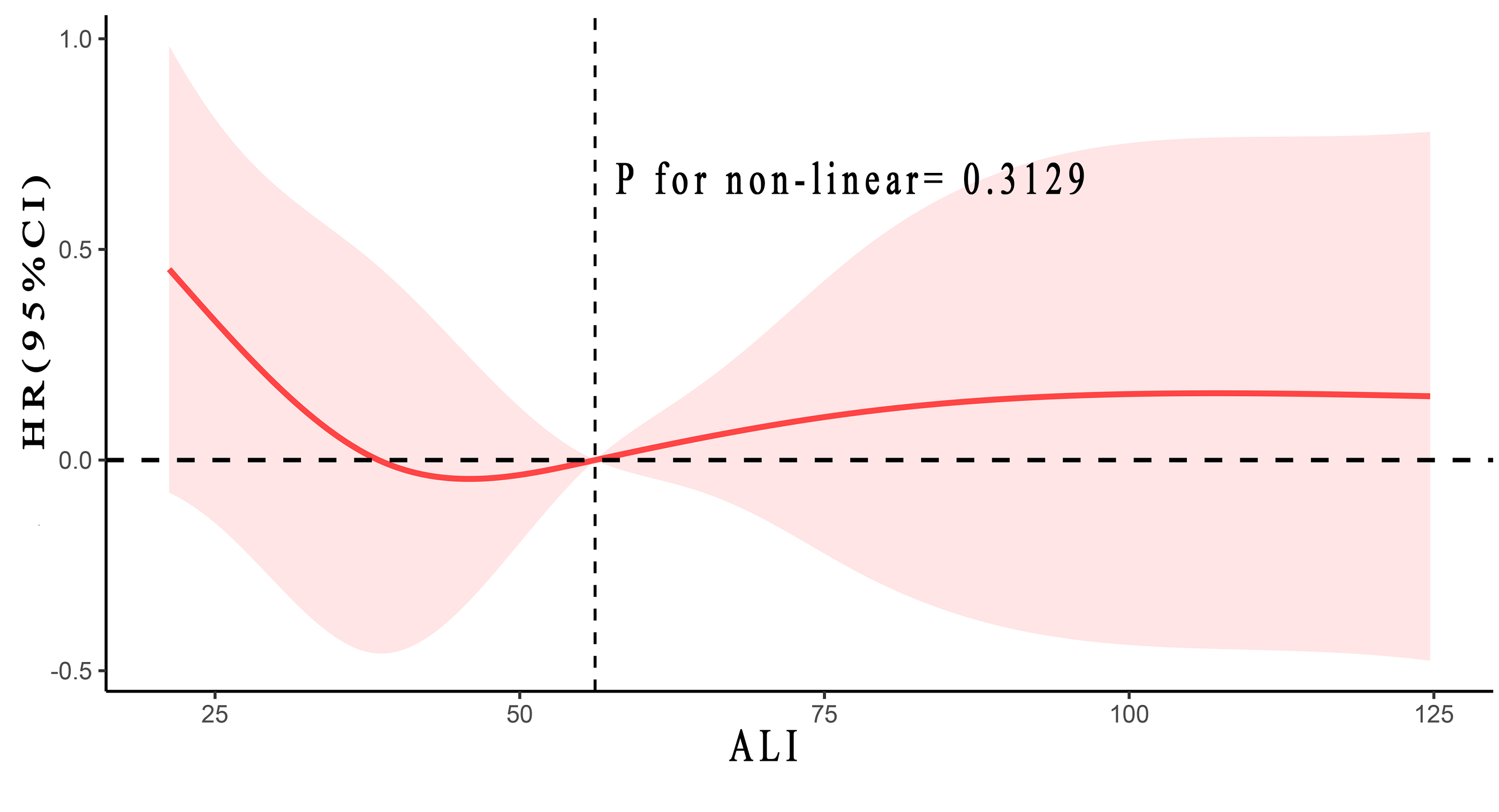


**Supplementary Figure S2.** Relationship between ALI and cancer mortality in patients with T2DM.


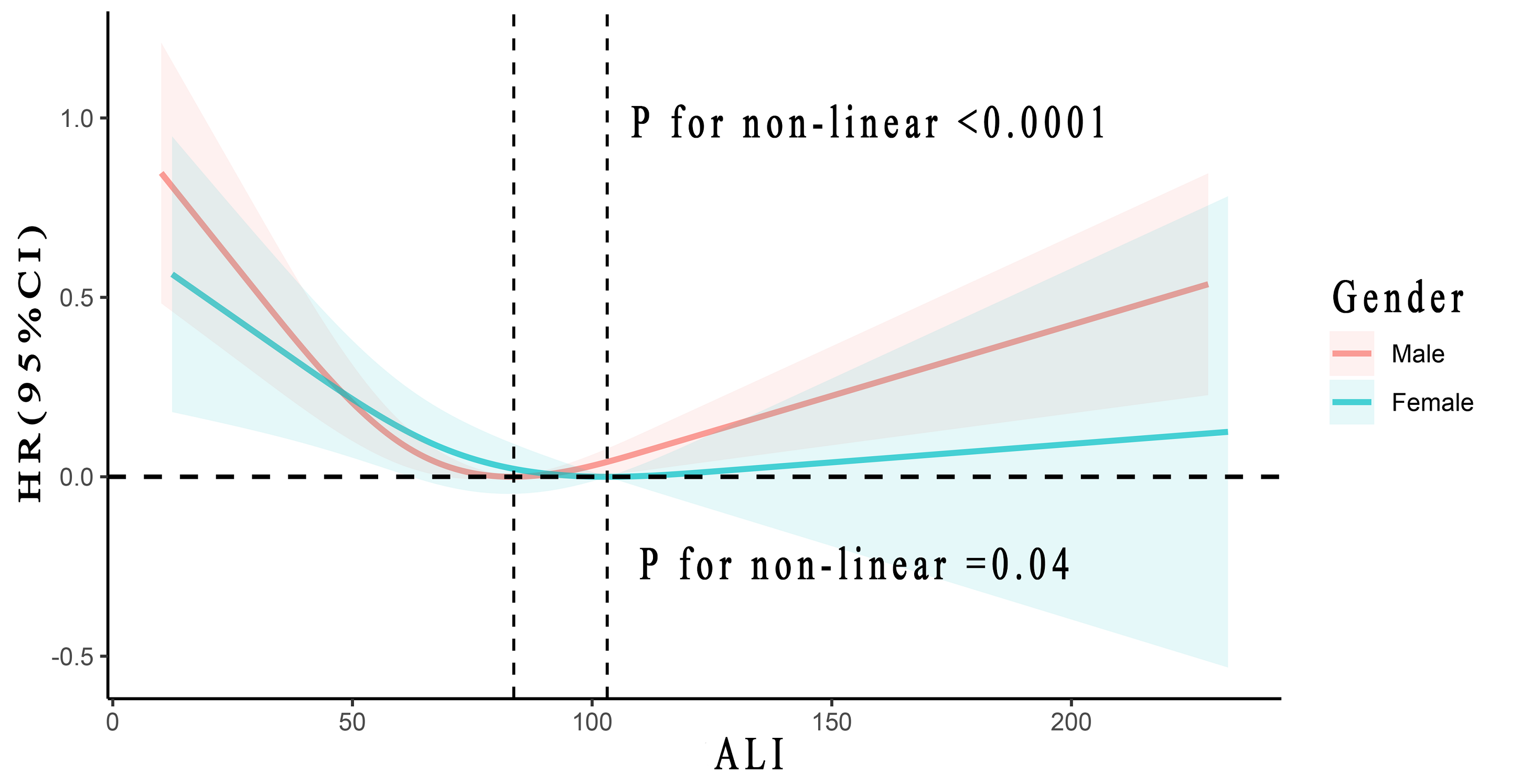


**Supplementary Figure S3.** Relationship between ALI and all-cause mortality stratified by gender in patients with T2DM.


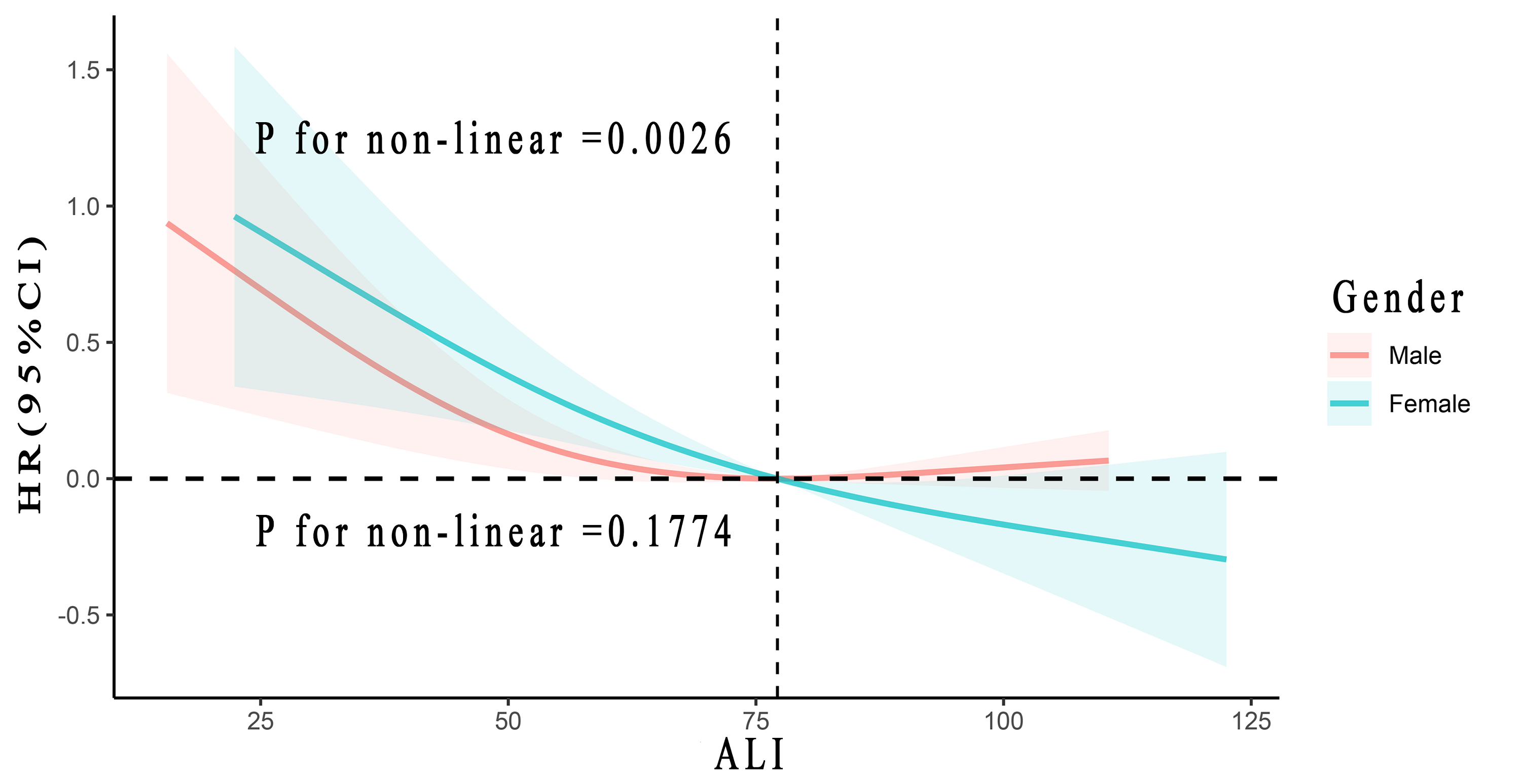


**Supplementary Figure S4.** Relationship between ALI and CVD mortality stratified by gender in patients with T2DM.
